# Prevalence and Presentation of Paediatric Coronavirus Disease 2019 in Lagos, Nigeria

**DOI:** 10.1155/2021/2185161

**Published:** 2021-10-06

**Authors:** Abideen Salako, Oluwatosin Odubela, Tomilola Musari-Martins, Priscilla Ezemelue, Titilola Gbaja-Biamila, Babasola Opaneye, Ayorinde James, Osaga Oforomeh, Kazeem Osuolale, Adesola Musa, Emelda Chukwu, Nurudeen Rahman, Agatha David, Rosemary Audu, Oliver Ezechi, Babatunde Salako

**Affiliations:** ^1^Nigerian Institute of Medical Research, Yaba, Lagos State, Nigeria; ^2^College of Medicine, University College Hospital, Ibadan, Oyo State, Nigeria

## Abstract

**Background:**

The objective of this study was to describe the prevalence and clinical features of coronavirus disease 2019 (COVID-19) among children (≤18 years) evaluated for Severe Acute Respiratory Syndrome Coronavirus 2 (SARS-CoV-2) infection at a testing centre in Lagos, Nigeria. *Methodology*. This was a retrospective study. Data on the sociodemographic, clinical characteristics and SARS-CoV-2 results of participants at a modified drive-through centre for COVID-19 test sample collection over four months were retrieved from the electronic medical records (EMR). Data obtained were analyzed using SPSS version 22.0.

**Results:**

A total of 307 children (≤18 years) were evaluated in this review. The prevalence of SARS-CoV-2 infection among the paediatric population was 16.3%. The median age (interquartile range (IQR)) was 9 (4–14) years. Common symptoms reported by the positive cases were fever (40.0%), cough (32.9%), sore throat (17.1%), and runny nose (15.7%). The majority of the positive cases had mild symptoms. Fever and sore throat were associated with the positive cases.

**Conclusion:**

Fever and sore throat were associated with SARS-CoV-2 infection among our cohort which buttresses the need for a high level of suspicion and clinical acumen in the management of common febrile diseases in paediatric settings.

## 1. Introduction

The current pandemic of the novel coronavirus disease 2019 (COVID-19) caused by the Severe Acute Respiratory Syndrome Coronavirus 2 (SARS-CoV-2) has ravaged the global community since it was first reported in the Hubei province, Wuhan, China, in December 2019. The disease has affected persons of all ages, with the elderly and individuals with underlying comorbid illness being most severely affected [1–3]. The ripple effects of the disease on global health and the economy warranted the World Health Organization (WHO) to declare it a public health emergency of international concern (PHEIC) in January 2020 [4, 5].

Nigeria, a nation with a population of over 200 million citizens (42% are within the paediatric age group) [6], has recorded over twenty-five thousand cases and five hundred and ninety mortalities from the disease as of June 2020 since the first confirmed case on 27th February 2020 [7]. Nigeria, like most African nations, has recorded fewer cases and deaths when compared to developed countries. However, most reports of the pandemic in the country are largely silent on the pattern and prevalence of the disease among the paediatric population. The few reports on SARS-CoV-2 among the paediatric population raise pertinent questions: “are children dying unrecognized” and “are the incidence and severity of COVID-19 truly low in the paediatric age group” [8–10]. The nonspecific nature of the disease and the similitude to other common childhood illnesses (ranging from acute respiratory tract infection (ARI), malaria, and acute diarrheal disease) could pose a challenge in the prompt diagnosis of SARS-CoV-2 infection in the paediatric population. This study is aimed at reviewing the data on screening for SARS-CoV-2 infection and thus describing the pattern of symptomatology and prevalence of COVID-19 among children tested for COVID-19 disease in Lagos, Nigeria.

## 2. Materials and Methods

This is a retrospective study describing the symptomatology and prevalence of COVID-19 among children at a testing centre. Data was collected from the electronic records of individuals visiting the modified drive-through SARS-CoV-2 testing centre, at the Nigerian Institute of Medical Research (NIMR), Lagos, from March 1, 2020, to June 30, 2020. The institute houses two national reference laboratories: the Centre for Tuberculosis Research (CTR) and the Centre for Human Virology and Genomics (CHVG). The CHVG is an ISO 15189-accredited laboratory involved in the diagnosis of viral infections and is also a WHO prequalification evaluating laboratory. The centre is a designated SARS-CoV-2 testing laboratory in the national network for COVID-19 laboratories.

A modified drive-through sample collection strategy was established for SARS-COV-2 testing at NIMR. The modalities of the registration and subsequent invitation to the centre have been described in a previous study [11]. The sociodemographic data (age, sex, place of and residence), travel history, preexisting comorbidities, clinical symptoms, and outcomes of the qualitative RT-PCR results (by Roche Cobas 6800) were extracted and analyzed using SPSS software, version 22.0 (IBM, Armonk, NY, USA). Categorical variables were presented as frequencies and percentages, while continuous variables were expressed as median (interquartile range). The chi-square or Fisher exact test was used to compare the differences between groups for categorical variables. Logistic regression (univariate and multivariate) was used to determine the association between study outcome and characteristics of participants. Results were considered statistically significant at *p* < 0.05.

The local health authorities (Nigeria Centre for Disease Control) have defined mild cases of COVID-19 as asymptomatic and those with nonspecific symptoms (fever, cough, sore throat, nasal congestion, malaise, headache, muscle pain, loss of smell, loss of taste, diarrhea, vomiting, and abdominal pain) [12]. BCG vaccination was assessed through visual inspection of BCG scar and reporting by caregivers. Recent travel history is defined as a voyage within the last 2 weeks from a country with a higher prevalence of COVID-19 cases which correlates with the incubation period for the disease.

The Institutional Review Board of the Nigerian Institute of Medical Research approved the study protocol. Obtained data were analyzed anonymously.

## 3. Results

**Figure 1:**
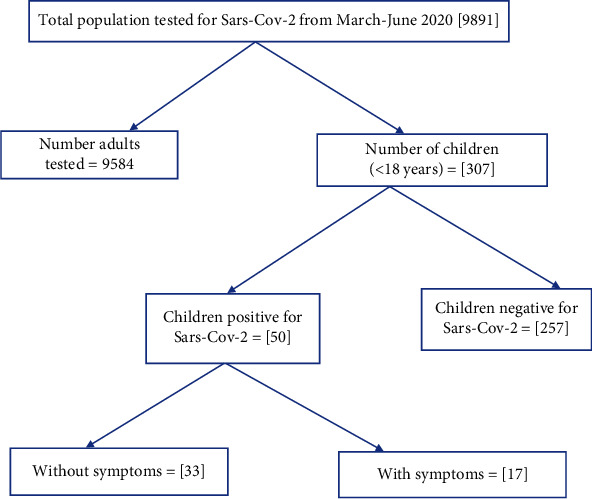
Flowchart of data review.

Of the 9891 clients screened at the NIMR centre during the period under review, 307 (3.1%) were children (<18 years). 50 children had positive results for SARS-CoV-2 giving a prevalence of 16.3%. Seventeen (34.0%) of the SARS-CoV-2-positive children presented with symptoms while the rest were asymptomatic (Figure 1).

**Figure 2:**
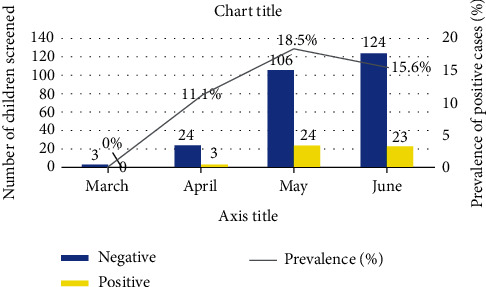
Pattern of children screened and outcomes.

The proportion of children screened increased over the study period, with an increasing trend in the positive cases from 0% in March, 12.5% in April, 18.5% in May and 15.6% in June, respectively (Figure 2).

The median (IQR) age of the children screened was 9 years (4–14 years). The majority were aged ≤9 years (53.1%), male (53.1%), had evidence of BCG vaccination (64.2%), and had recently travelled to a country with high COVID-19 incidence or come in contact with an individual with confirmed SARS-CoV-2 infection (59%). Preexisting medical conditions were present in 5.5%, and these included asthma (3.9%, 12/307), congenital heart disease (0.9%, 3/307), and obesity (0.7%, 2/307) (Table 1).

**Figure 3:**
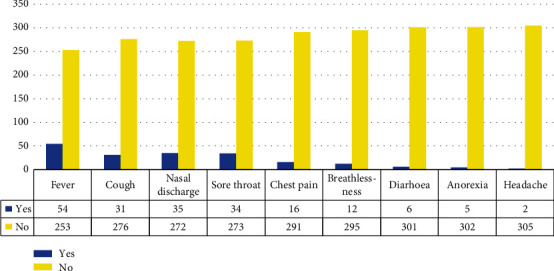
Symptomatology of the children screened for SARS-CoV-2.

The most common symptoms among the children screened for SARS-CoV-2 were fever (17.6%), cough (10.1%), runny nose (11.4%), sore throat (11.1%), and chest pain (5.2%) Figure 3.

The median (IQR) age was 9 (4–14) years. There were 162 males and 145 females, giving a male : female ratio of 1.1 : 1. A majority had a positive history of BCG vaccination (64.2%), contact with confirmed COVID-19 case or travel (59.0%), and no preexisting medical condition (94.5%). One hundred and twenty-six participants (41.0%) presented with one or more symptoms. Fifty participants tested positive by RT-PCR to SARS-CoV-2 giving a prevalence of 16.3%. There was no statistically significant difference in study variables among SARS-CoV-2-positive and SARS-CoV-2-negative children. The age, gender, history of contact, BCG vaccinations, and symptomatology at presentation had no significant association with SARS-CoV-2 infection among the children screened (Table 2).

Fever, sore throat, and chest pain were significantly associated with being SARS-CoV-2 positive following univariate analysis, but this association was not evident with multivariate analysis (Table 3). The two reports of headache were found only among SARS-CoV-2-positive persons.

## 4. Discussion

The COVID-19 pandemic has had an impact on the continent. However, most information on the disease is focused on the adult population with limited information about the burden and pattern of the disease in the paediatric population, especially in sub-Saharan Africa.

The prevalence of SARS-CoV-2 infection among the paediatric population in our study (16.3%) is within the prevalence range globally (0.39%–34.1%). Similar studies done within the same time frame as the current study reported prevalence between 0.39% and 34.1% [3, 8, 13–16].

The increase in the number of children screened for the COVID-19 and the subsequent rise in the prevalence from zero to 18.5% in the current study could be explained by the mandatory national policy of the need to screen close contacts of positive cases as well as Lagos state being the epicentre for the infection in the country. This finding affirms the human-to-human transmission and is similar to the trend reported by Dong et al. [16] in China.

Most of the children with COVID-19 disease in our study were asymptomatic or had mild symptoms, which is in keeping with previous reports [8, 16–20]. The plausible explanations for this presentation among children aside from being a drive-through testing centre may be the robust innate immune system, weaker adaptive immune response, viral containment, or clearance in children [9, 10, 21–24]. Other postulations include recurrent exposure of children to viral infections modulating their responses to SARS-CoV-2 [9, 10], the high level of melatonin in children in addition to its anti-inflammatory and oxidative properties (inhibiting SARS-CoV-2 infection through the blockage of the CD147 receptor) [25–27], exposure to live attenuated vaccine (BCG, OPV) [28–31], and low expression and function of the angiotensin 2 (ACE2) receptors [32, 33].

The pattern of symptomatology (fever, sore throat, cough, chest pain, and other gastrointestinal symptoms) among the screened population and confirmed SARS-CoV-2 infection is similar to the case report series by Ibrahim et al. [34] in Nigeria. However, none of the cases reported by Ibrahim and his colleagues had gastrointestinal symptoms [34]. Furthermore, the pattern of symptoms in our review aligns with the clinical symptomatology of COVID-19 reported in children by Rahimzadeh et al. [35] in Iran, Le et al. [36] in Vietnam, the CDC COVID response team in the United States of America [8], and various other reports describing the symptomatology of the disease in the paediatric population [17–20]. This symptomatology of the SARS-CoV-2 infections and its similarity to common childhood infections such as malaria, acute diarrhea diseases, and respiratory tract infection buttress the need for a high index of suspicion to ensure early diagnosis and prompt treatment.

There was slightly higher proportion of males infected with SARS-CoV-2, though without any significant difference noted with regard to gender and COVID-19 infection in the current study. The increased male predominance might be coincidental but could also be attributable to the increased ACE2 expression caused by testosterone in males as against the reversal effects by estrogens in females. Several previous studies have shown that the sex hormones androgens and estrogens influence the renin-angiotensin system. Androgens increase plasma renin activity and expression of angiotensinogen messenger RNA, while estrogens decrease plasma renin activity and angiotensin receptor expression [37–39]. This could explain the male predominance among COVID-19 cases, though this hypothesis requires further evaluation in the affected population across all the age groups. The male predominance among the SARS-CoV-2-infected children aligns with reports by CDC in USA [8], Guan et al. [18], and Dong et al. [16].

Contrary to reports of the association between age, BCG vaccination status, comorbid illness, and the COVID-19 infection in previous studies [8, 18], our findings did not show any association between the infection and the above-listed variables. This could be because our facility is only a testing centre; other clinical data or extensive epidemiological review might proffer better clarity. However, the association of comorbid illness and BCG vaccination status requires more evidence-based findings beyond the scope of this report. The paediatric population at the centre appeared evenly distributed except for children aged below 12 months. This is not surprising considering the nature of our services contrary to other studies from hospitals or epidemiological data [8, 16–18].

This study is the first to describe the epidemiological and clinical characteristics of children screened for SARS-CoV-2 infection in sub-Saharan Africa. The clinical outcome of positive cases could not be ascertained as that data was not available to us. In addition, there could be information bias to the symptoms declared by caregivers on behalf of their wards.

## 5. Conclusions

Fever and sore throat were associated with positive COVID-19 results. The symptomatology of infected children is similar to other common childhood illnesses in the country (ARI, acute diarrhea diseases, viral infection, and malaria). A high index of suspicion is needed to combat the pandemic in the paediatric population among other health-promoting and preventive measures.

## Figures and Tables

**Table 1:** Characteristics of the children screened for SARS-CoV-2 infection.

Characteristics	Patients (%)
Age group (years)	
<1	6 (2.0)
1–4	75 (24.4)
5–9	82 (26.7)
10–14	70 (22.8)
>14	74 (24.1)
Sex	
Female	144 (46.9)
Male	163 (53.1)
Nationality	
Nigerian	277 (90.2)
Non-Nigerian	30 (9.8)
Preexisting medical condition	
No	290 (94.5)
Yes	17 (5.5)
History of contact/travel	
Yes	181 (59.0)
No	126 (41.0)
BCG vaccination	
Yes	197 (64.2)
No/unknown	110 (35.8)
Symptomatic	
Yes	126 (41.0)
No	181 (59.0)
SAR-CoV-2 test result	
Positive	50 (16.3)
Negative	257 (83.7)

**Table 2:** Association between screening outcome and participants' characteristics.

Characteristics	All (*n* = 307)	SARS Pos (*n* = 50)	SARS Neg (*n* = 257)	Univariate		Multivariate	
				OR [95% CI]	*p* value	OR [95% CI]	*p* value
Median age (IQR) (years)	9.0 (4.0–14.0)	10.0 (5.3–15.0)	9.0 (4.0–14.0)				
Age group (years)							
<10	163 (53.1)	22 (44.0)	141 (54.9)	1.55[0.87–2.85]	0.159	1.56[0.83–2.91]	0.165
≥10	144 (46.9)	28 (56.0)	116 (45.1)				
Sex							
Male	162 (52.8)	27 (54.0)	135 (52.5)	1.06[0.58–1.95]	0.849	1.09[0.59–2.02]	0.786
Female	145 (47.2)	23 (46.0)	122 (47.5)				
BCG							
Yes	197 (64.2)	32 (64.0)	165 (64.2)	0.99[0.59–1.68]	0.978	0.99[0.51–1.94]	0.986
No/not sure	110 (35.8)	18 (36.0)	92 (35.8)				
Contact/travel history							
Yes	181 (59.0)	31 (62.0)	150 (58.4)	1.16[0.62–2.17]	0.632	1.15[0.59–2.24]	0.689
No	126 (41.0)	19 (38.0)	107 (41.6)				
Preexisting condition							
Yes	17 (4.5)	1 (2.0)	16 (6.2)	0.31[0.04–2.37]	0.232	0.34[0.04–2.74]	0.313
No	290 (94.5)	49 (98.0)	241 (93.8)				
Symptoms							
Yes	126 (41.0)	17 (34.0)	109 (42.4)	0.70[0.37–1.32]	0.269	0.70[0.37–1.34]	0.283
No	181 (59.0)	33 (66.0)	148 (57.6)				

**Table 3:** Comparison of symptoms among SARS-positive and SARS-negative participants.

Symptoms	All (*n* = 126)	SARS Pos (*n* = 17)	SARS Neg (*n* = 109)	Univariate		Multivariate	
				OR [95% CI]	*p* value	OR [95% CI]	*p* value
Fever							
Yes	54 (42.8)	12 (70.6)	42 (38.5)	3.82[1.26–11.64]	0.013	0.49[0.12–1.98]	0.314
No	72 (57.2)	5 (29.4)	67 (61.5)				
Cough							
Yes	31 (24.6)	5 (29.4)	26 (23.8)	1.33[0.43–4.13]	0.621	0.50[0.12–2.06]	0.337
No	95 (75.4)	12 (70.6)	83 (76.2)				
Runny nose							
Yes	35 (27.8)	4 (23.5)	31 (28.4)	0.77 [0.23–2.56]	0.674	0.35 [0.08–1.44]	0.144
No	91 (72.2)	13 (76.5)	78 (71.6)				
Sore throat							
Yes	34 (27.0)	9 (52.9)	25 (22.9)	3.78[1.32–10.82]	0.009	4.59[0.93–22.73]	0.062
No	92 (73.0)	8 (47.1)	84 (77.1)				
Chest pain							
Yes	16 (12.7)	5 (29.4)	11 (10.1)	3.71[1.10–12.51]	0.026	2.03[0.43–9.69]	0.374
No	110 (87.3)	12 (70.6)	98 (89.9)				
Breathing difficulty							
Yes	12 (9.5)	2 (11.8)	10 (9.2)	1.32 [0.26–6.62]	0.665	0.26 [0.03–2.04]	0.201
No	114 (94.5)	15 (88.2)	99 (90.8)				
Diarrhea							
Yes	6 (4.8)	2 (11.8)	4 (3.7)	3.5[0.59–20.79]	0.186	10.56[0.73–152.93]	0.084
No	120 (95.2)	15 (88.2)	105 (96.3)				
Anorexia							
Yes	5 (4.0)	1 (5.9)	4 (3.7)	1.64[0.17–15.62]	0.522	20.31[0.56–726.39]	0.099
No	121 (96.0)	16 (94.1)	105 (96.3)				

## Data Availability

Access to this data is restricted due to the sensitive nature and stigma associated with COVID-19 in our setting.
